# Improving Striae Distensae With Combined Hyperdiluted Calcium Hydroxylapatite Injection and Fractional Microneedling Radiofrequency: A Pilot Study

**DOI:** 10.1093/asjof/ojag121

**Published:** 2026-07-09

**Authors:** Haitham M Saleh, Daniella Fakih, Ahmed Elbeltagy, Sham AlZahabi, Hossam Elenany, Lorena Arrien, Nabil Fakih-Gomez

## Abstract

*Striae distensae* (SD), or stretch marks, are dermal scars resulting from disrupted collagen and elastin because of mechanical stress. Effective treatments, particularly for chronic striae alba, remain limited. Calcium hydroxylapatite (CaHA), a biostimulatory dermal filler, and fractional microneedling radiofrequency (RF) have individually demonstrated regenerative effects. This pilot study investigates the safety and efficacy of combining CaHA and fractional microneedling RF in improving SD. Eight female patients with abdominal SD (rubra or alba) underwent 1 session of hyperdiluted CaHA injection (1:4 dilution, 3 mL), followed by 3 monthly sessions of RF microneedling. Outcomes were evaluated 6 months after the final session using the Manchester Scar Scale (MSS), patient satisfaction scores, and adverse event monitoring. All participants (mean age 36.6 ± 7.3 years) completed the protocol. Mean MSS scores improved significantly from 14 ± 0.93 at baseline to 6.88 ± 1.64 posttreatment (*P* < .0001). The mean satisfaction score was 8.12 ± 1.72. Mild, transient erythema occurred in 2 cases; no serious adverse events were observed. This pilot study suggests that combining hyperdiluted CaHA with RF microneedling is a safe and effective approach for treating SD, yielding both clinical and patient-reported improvements. Larger controlled studies are warranted.

Level of Evidence: 4 (Therapeutic)

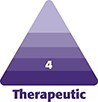

*Striae distensae* (SD), or stretch marks, are linear, atrophic dermal scars that result from tearing of the dermis because of rapid mechanical stretching. They are common in both sexes but occur more frequently in females.^1^ SD typically develops during adolescence, pregnancy (striae gravidarum), significant weight changes, endocrine disorders, or prolonged corticosteroid use.^2^

SD progresses from an initial inflammatory phase, known as striae rubra (SR), which presents as erythematous or violaceous lesions, to a chronic phase, striae alba (SA), characterized by hypopigmented and atrophic scars.^3^ Histological findings include epidermal thinning and disruption of collagen and elastin networks.^4^ Although not medically harmful, SD can significantly affect self-esteem and quality of life, especially in women of reproductive age.^5^

The pathogenesis of SD is complex and involves hormonal influences, genetic factors, and mechanical stress that compromises fibroblast activity and alters the dermal extracellular matrix.^6^ Current treatments such as topical agents, peels, microneedling, and laser therapies have yielded variable results, particularly in managing mature SA.^3^

Calcium hydroxylapatite (CaHA) is a biocompatible and biodegradable dermal filler composed of synthetic calcium and phosphate ions suspended in a carboxymethylcellulose gel.^7^ Initially used in reconstructive procedures, it is now widely applied in facial rejuvenation and regenerative treatments.^8^ Beyond its volumizing effect, CaHA stimulates fibroblasts to promote neocollagenesis and elastin production.^9,10^ When diluted with saline and lidocaine, the hyperdiluted form allows diffuse biostimulation without bulk formation.^11,12^

CaHA influences dermal remodeling by increasing production of Collagen types I and III, enhancing angiogenesis, and improving skin thickness and elasticity.^13,14^ Its effectiveness is further enhanced when combined with technologies such as microneedling, radiofrequency (RF), or laser devices.^15^ Careful technique and patient selection are necessary to avoid adverse effects.^7^

Fractional microneedling RF is a minimally invasive method that combines controlled epidermal perforation with RF energy to stimulate tissue regeneration.^16,17^ By creating targeted thermal zones within the dermis, RF microneedling activates fibroblasts and supports new collagen and elastin formation without damaging the superficial skin layers.^18,19^ Clinical studies have shown visible improvement in both SR and SA, with better texture, reduced lesion depth, and high patient satisfaction.^20,21^

To date, no studies have evaluated the combined use of hyperdiluted CaHA and RF microneedling in treating SD.^22^ Their distinct yet complementary regenerative mechanisms may offer synergistic effects. This pilot study aims to investigate the safety and efficacy of this combination for improving the appearance of abdominal striae.

## METHODS

### Study Design and Population

This prospective, single-arm pilot study was conducted in accordance with the ethical principles of the Declaration of Helsinki. Informed consent was obtained from all participants before enrollment. Eligible participants included female patients aged 25 to 45 years presenting with SD (either rubra or alba) localized to the abdominal region. All participants had Fitzpatrick skin types I through IV. Exclusion criteria included current pregnancy or lactation, active skin infections, autoimmune disease, or recent use of systemic or topical retinoids or corticosteroids.

### Treatment Protocol

Each patient underwent a total of 4 treatment sessions, spaced 1 month apart. The first session consisted of an injection of hyperdiluted CaHA (Radiesse; Merz Aesthetics, Frankfurt, Germany), whereas sessions 2 through 4 involved fractional RF microneedling.

In the initial session, CaHA was diluted in a 1:4 ratio using equal parts of 2% lidocaine and normal saline (1.5 mL CaHA + 6 mL diluent [saline/lidocaine] = 7.5 mL total). We utilized a Luer–Lock connector to transfer the filler and diluent (lidocaine/saline) between syringes a minimum of 20 times to guarantee homogeneity. A total volume of 3 mL of the diluted filler was administered subdermally in the periumbilical region, targeting the affected SD, using the linear threading technique and a 25-gauge cannula. The CaHA injections were followed by rigorous massage of the region.

For the second through fourth sessions, fractional RF microneedling was performed using a device (Morpheus 8; InMode, Yokneam, Israel) equipped with a 40-needle cartridge. Each session involved 2 passes. The first pass was delivered in burst mode at a depth of 7 mm and an energy level of 25. The second pass was applied in cycle mode at a depth of 2 mm and an energy level of 20. A topical anesthetic containing 5% lidocaine was applied to the treatment area 30 min before each session to ensure patient comfort. Following each treatment session, the region was sanitized, and a plastic bandage was applied for a duration of 4 h. Subsequently, patients were instructed to refrain from sun exposure and to apply an emollient cream for 1 week.

### Evaluation Methods

Clinical outcomes were assessed using standardized digital photography and objective scar assessment tools. Pretreatment and 6-month posttreatment photographs were taken under identical lighting and positioning conditions.

Two board-certified dermatologists independently evaluated the pre- and post-6-month photographs utilizing the Manchester Scar Scale (MSS), with their scores averaged and subsequently compared.^23^ The MSS evaluates parameters such as color, texture, contour, and distortion Supplemental Figure 1. Patient-reported outcomes were measured using a 10-point Likert scale to rate satisfaction with the treatment results.

### Adverse Events Monitoring

All participants were monitored during and after each session for adverse effects, including erythema, edema, infection, and scarring. Any complications were documented throughout the study period.

### Statistical Analysis

Paired *t*-tests were used to compare the average pretreatment and posttreatment MSS scores. A *P*-value of <.05 was considered statistically significant. Descriptive statistics were employed to summarize patient satisfaction scores and the incidence of adverse events.

## RESULTS

**Figure 1.**
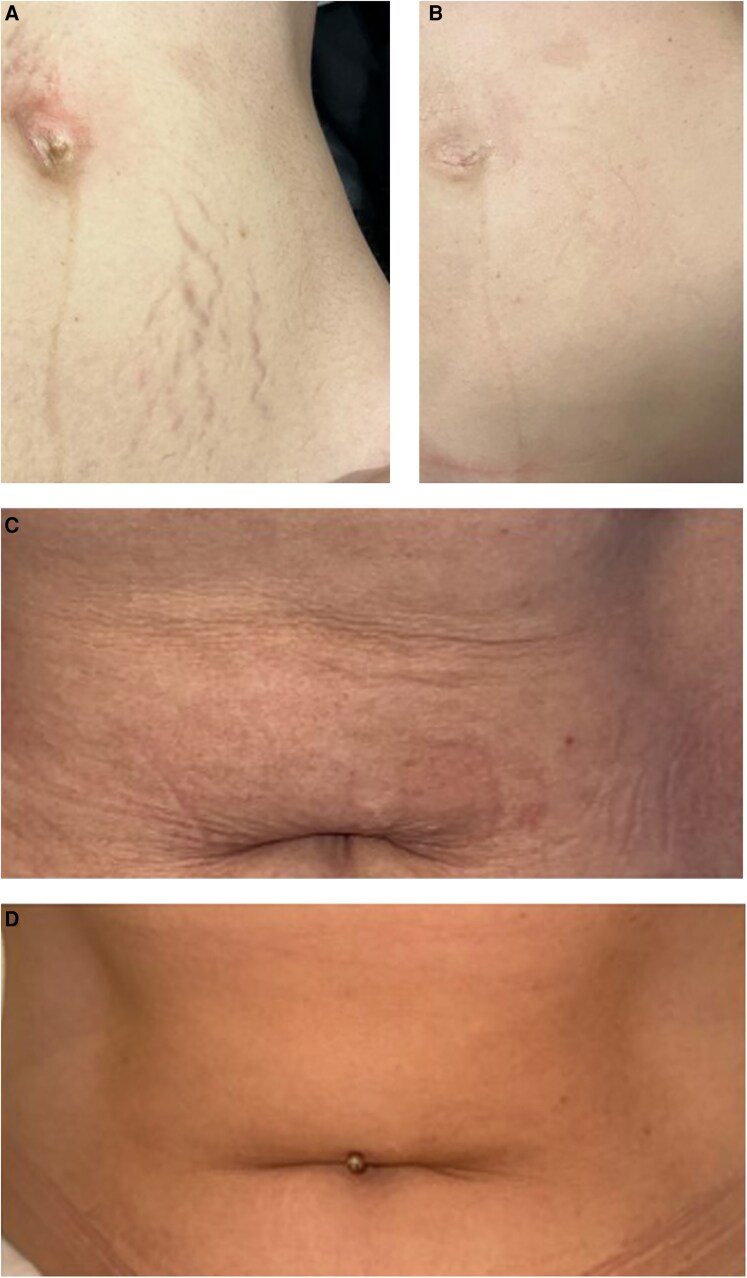
Before and after of patients with stria rubra. (A) A 31-year-old woman (Patient 1) with striae rubra, average Manchester Scar Scale (MSS) = 13 (obvious color mismatch, shiny finish, slightly raised, with moderate distortion and firm texture). (B) Six months after treatment of Patient 1, average MSS = 5 (perfect color match, matte finish, smooth contour blending with the surrounding skin, no distortion, and normal texture). (C) A 48-year-old woman (Patient 2) with striae rubra, average MSS = 11.5 (slight color mismatch, shiny finish, slightly raised, with mild/moderate distortion and firm texture). (D) Six months after completing the treatment protocol of Patient 2, average MSS = 5 (no distortion and smooth contour blending with the surrounding skin).

**Figure 2.**
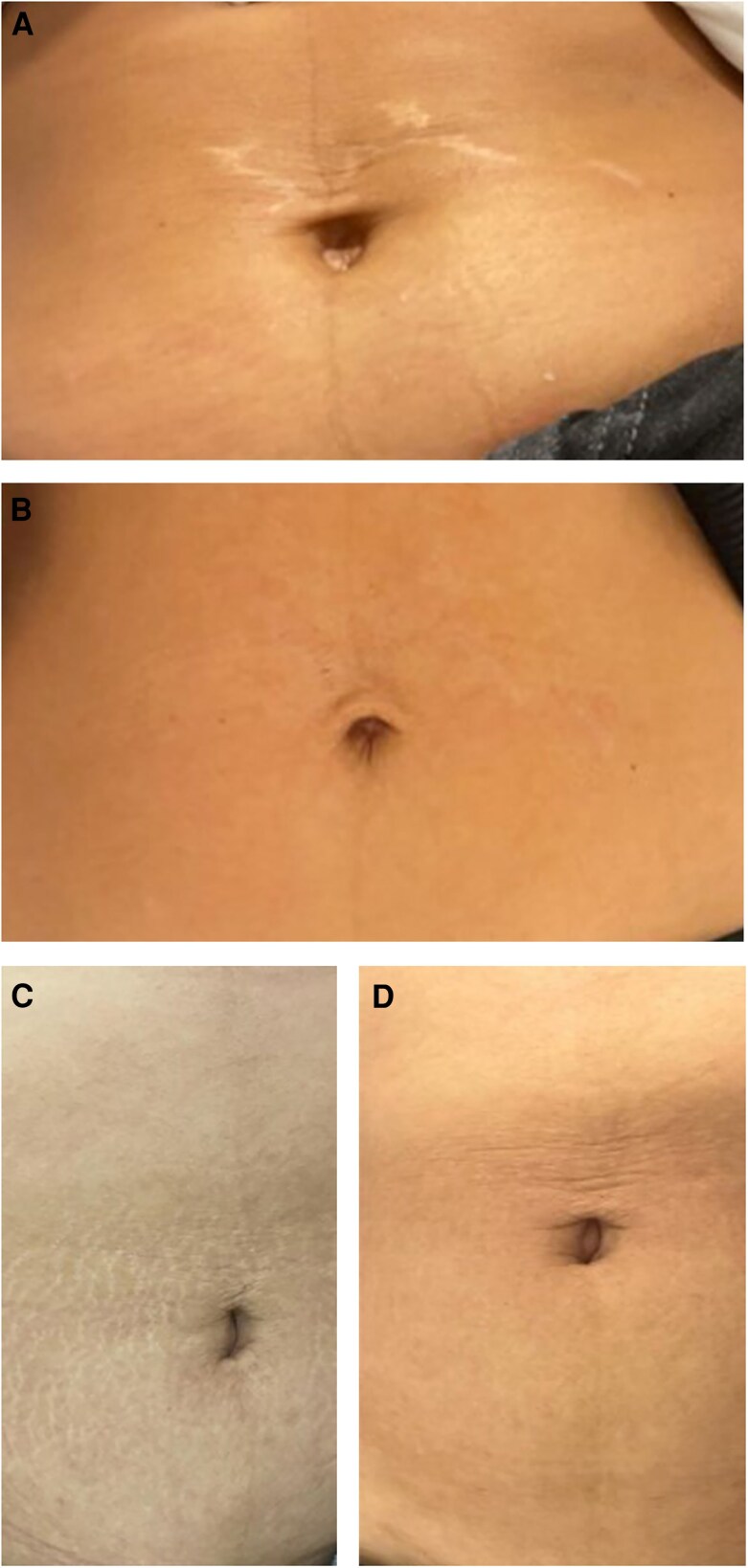
Before and after of patients with stria alba. (A) A 39-year-old woman (Patient 3) with irregular linear striae alba above the umbilicus, average Manchester Scar Scale (MSS) = 15.5 (gross color mismatch, shiny finish, slightly raised, with moderate/severe distortion and firm texture). (B) Six months after completing the treatment protocol of Patient 3, average MSS = 5 (no distortion and smooth contour blending with the surrounding skin). (C) A 47-year-old woman (Patient 4) with irregular linear striae alba, average MSS = 13.5 (slight/obvious color mismatch, shiny finish, slightly raised, with moderate distortion and hard texture). (D) Six months after treatment of Patient 4, MSS = 5.5 (perfect/slight color mismatch, matte finish, flush with the surrounding skin, no distortion, and normal texture).

A total of 8 female patients successfully completed the treatment protocol, with a mean age of 36.6 ± 7.3 years. Among them, 3 presented with SR, the early erythematous stage, and 5 with SA, the chronic hypopigmented form (Figures 1, 2). All lesions were localized to the periumbilical abdominal region, an area commonly affected by dermal stretching, particularly following pregnancy or significant weight fluctuations. Treatment efficacy was assessed using the MSS, which evaluates scar characteristics, including color, contour, and texture. The mean baseline MSS score was 14 ± 0.93, indicating moderate-to-severe dermal atrophy. After completion of the 4-session protocol, the mean MSS score was significantly reduced to 6.88 ± 1.64, representing a mean reduction of 7.12 points (±1.43), with a *P*-value of <.0001, confirming statistical significance. Patient-reported satisfaction measured using a 10-point Likert scale (1 = extreme dissatisfaction, 10 = maximum satisfaction), yielded a mean score of 8.12 ± 1.72. Six out of eight patients rated their satisfaction as 8 or higher, reflecting a high level of approval regarding the aesthetic improvement. In terms of safety, no serious adverse events were reported. Two patients experienced mild, transient erythema lasting 24 to 48 h, and no cases of infection, nodule formation, or postinflammatory hyperpigmentation were observed.

## DISCUSSION

SD are a complex dermatological condition associated with significant aesthetic and emotional consequences, particularly among women. The transition from the early inflammatory phase to the chronic atrophic stage indicates progressive structural damage within the dermis. This condition arises from a combination of hormonal fluctuations, mechanical stress, and genetic predisposition, which together disrupt fibroblast function and compromise the extracellular matrix.^6^ Although a variety of therapeutic approaches exist, including topical agents, chemical peels, microneedling, and laser technologies, treatment outcomes remain inconsistent, especially in the more resistant SA subtype.^3^

CaHA is a biocompatible and biodegradable dermal filler that has gained widespread use in both aesthetic and reconstructive dermatology. It consists of synthetic calcium and phosphate ions, structured as microspheres suspended in a carboxymethylcellulose gel. This composition closely mimics the mineral content of human bone, contributing to its high tolerability and minimal immunogenicity.^7^ Initially designed for applications such as vocal cord augmentation and correction of soft tissue defects, CaHA is now commonly used in facial volumization and skin rejuvenation procedures.^8^

Beyond its volumizing role, CaHA demonstrates notable biostimulatory effects. Once injected, the gel carrier provides immediate but temporary volume, whereas the CaHA microspheres remain in the dermis and stimulate fibroblast activity. This promotes neocollagenesis and the synthesis of elastin, contributing to gradual tissue remodeling.^9^ This dual action, which includes both instant correction and long-term regenerative effects, sets CaHA apart from fillers that function solely by increasing volume.^10^

Recent innovations in application techniques have expanded the utility of CaHA. When diluted with saline and lidocaine, the product, referred to as hyperdiluted CaHA, loses its volumizing capacity but retains its ability to stimulate collagen synthesis. This form allows for broader and more uniform application, making it suitable for larger treatment areas with reduced risk of nodule formation or excessive tissue volume.^11^ Hyperdiluted CaHA has been effectively applied to enhance skin quality in areas such as the neck, décolleté, arms, thighs, and buttocks.^12^

The regenerative capacity of CaHA is mediated by mechanical and biochemical pathways that influence extracellular matrix remodeling. It increases the production of Type I and Type III collagen, stimulates angiogenesis and elastogenesis, and results in improved dermal thickness and skin texture.^13^ These properties make CaHA especially beneficial for conditions involving dermal atrophy and structural compromise, including photoaging, skin laxity, and atrophic scarring.^14^

Because interest in nonsurgical skin regeneration grows, hyperdiluted CaHA has emerged as a promising agent in combination therapies. Its capacity to enhance the results of procedures such as microneedling, RF, and laser treatments is well documented, showing synergistic effects in promoting dermal renewal.^15^ Although generally safe, optimal outcomes depend on proper technique and patient selection, as adverse effects such as bruising, swelling, and in rare cases, nodule formation or vascular complications may occur. When applied correctly, CaHA provides a reliable option for both structural support and tissue regeneration.^7^

RF is a noninvasive method increasingly used in dermatology and aesthetic medicine for dermal remodeling through controlled thermal induction.^16^ This technology combines mechanical skin penetration with RF energy delivery to stimulate regenerative processes within different layers of the skin. It has been particularly successful in the treatment of conditions involving dermal atrophy, such as acne scars, photoaging, and SD.^17^

RF microneedling devices consist of fine needles that penetrate the epidermis and deliver RF energy directly into the dermis. This process generates heat within targeted layers, activating biological pathways without significantly harming the surface skin.^18^ These thermal microzones promote fibroblast activation and increase the production of Type I and Type III collagen and elastin. The depth and intensity of stimulation can be precisely tailored by adjusting needle length and energy output, allowing for individualized treatment strategies.^19^

This technique offers several advantages in the management of striae. The thermal energy improves dermal density and elasticity, whereas the microneedling component facilitates epidermal turnover and pigment regulation. The fractional application method promotes rapid healing and minimizes downtime, making the treatment suitable for various skin types and anatomical areas.^20^ Clinical studies have reported improvements in both SR and SA following RF microneedling, with better skin texture, reduced lesion dimensions, and high levels of patient satisfaction.^21^

In response to the challenges of treating SD, combined approaches that integrate different therapeutic mechanisms are gaining popularity. The use of fractional RF microneedling together with injectable biostimulators, such as hyperdiluted CaHA, is increasingly recognized for its ability to deliver enhanced outcomes.^22^

The findings of this pilot study support the effectiveness and safety of combining hyperdiluted CaHA with fractional microneedling RF for the aesthetic treatment of SD. The significant reduction in MSS scores observed after treatment indicates meaningful improvement in scar morphology, skin tone, and overall texture. Patient satisfaction was high, as demonstrated by Likert scale responses, reinforcing the cosmetic benefit and tolerability of this combination therapy.

This clinical improvement can be explained by the distinct yet complementary mechanisms of each treatment. Hyperdiluted CaHA functions as a potent biostimulatory agent that activates fibroblasts and enhances the production of collagen, elastin, and extracellular matrix components. The result is gradual dermal thickening and increased skin elasticity, with visible reduction in the atrophic appearance of stretch marks. The absence of added volume makes this approach particularly appropriate for treating large or diffuse areas such as the abdominal region.^7,14,24^

At the same time, fractional microneedling RF contributes to dermal remodeling through the creation of controlled thermal zones within the skin. These zones trigger a localized wound-healing cascade that includes fibroblast activation, release of growth factors, collagen remodeling, and angiogenesis.^25,26^ In this study, the use of a dual-pass RF technique with different energy settings and needle depths enabled comprehensive stimulation of both superficial and deep dermal layers.

The synergistic interaction between these 2 treatments is particularly effective. CaHA supports the biochemical environment necessary for sustained tissue regeneration, whereas RF microneedling provides immediate stimulation for tightening and matrix remodeling.^27^ Together, they address both the structural deficits and surface irregularities associated with SR and SA, achieving results that may not be attainable with a single modality.^21^

Adverse effects were minimal, limited to transient erythema in 2 participants. This outcome is consistent with previous research supporting the safety of both treatments when applied with appropriate technique and clinical judgment.^28,29^

Nevertheless, this study has certain limitations. The small sample size reduces statistical strength and generalizability. The absence of a control or comparison group prevents clear attribution of efficacy to the individual components of the combined treatment. Additionally, the follow-up period was relatively short, which limits insight into the long-term durability of the results. Although the MSS provides valuable clinical data, histological analysis was not performed and would have strengthened the evidence of dermal remodeling and neocollagenesis.

Despite these constraints, this pilot study offers valuable preliminary insight into a combination approach for treating SD. Given the resistance of conditions such as SA to conventional therapies, strategies that simultaneously activate mechanical and biochemical pathways appear especially promising. Future studies should aim to refine treatment parameters, compare single and combined interventions, and include longer follow-up periods and histological evaluation to assess sustained efficacy.

## CONCLUSIONS

This pilot study provides preliminary evidence that the combination of hyperdiluted CaHA and fractional RF microneedling may be a safe, well tolerated, and clinically effective treatment for improving the appearance of SD. Both objective evaluations and subjective patient-reported outcomes support the efficacy of this multimodal approach in enhancing skin texture and elasticity. Although the results are encouraging, larger randomized controlled trials incorporating histological analysis and extended follow-up are needed to validate these findings and establish standardized treatment protocols for this prevalent and therapeutically challenging dermatologic condition.

## Supplementary Material

ojag121_Supplementary_Data
