# Supplementary figures and images for: Improving Striae Distensae With Combined Hyperdiluted Calcium Hydroxylapatite Injection and Fractional Microneedling Radiofrequency: A Pilot Study
Source: Aesthet Surg J Open Forum. 2026 Jul 9;8:ojag121. doi: 10.1093/asjof/ojag121 (PMC13353822; doi:10.1093/asjof/ojag121)

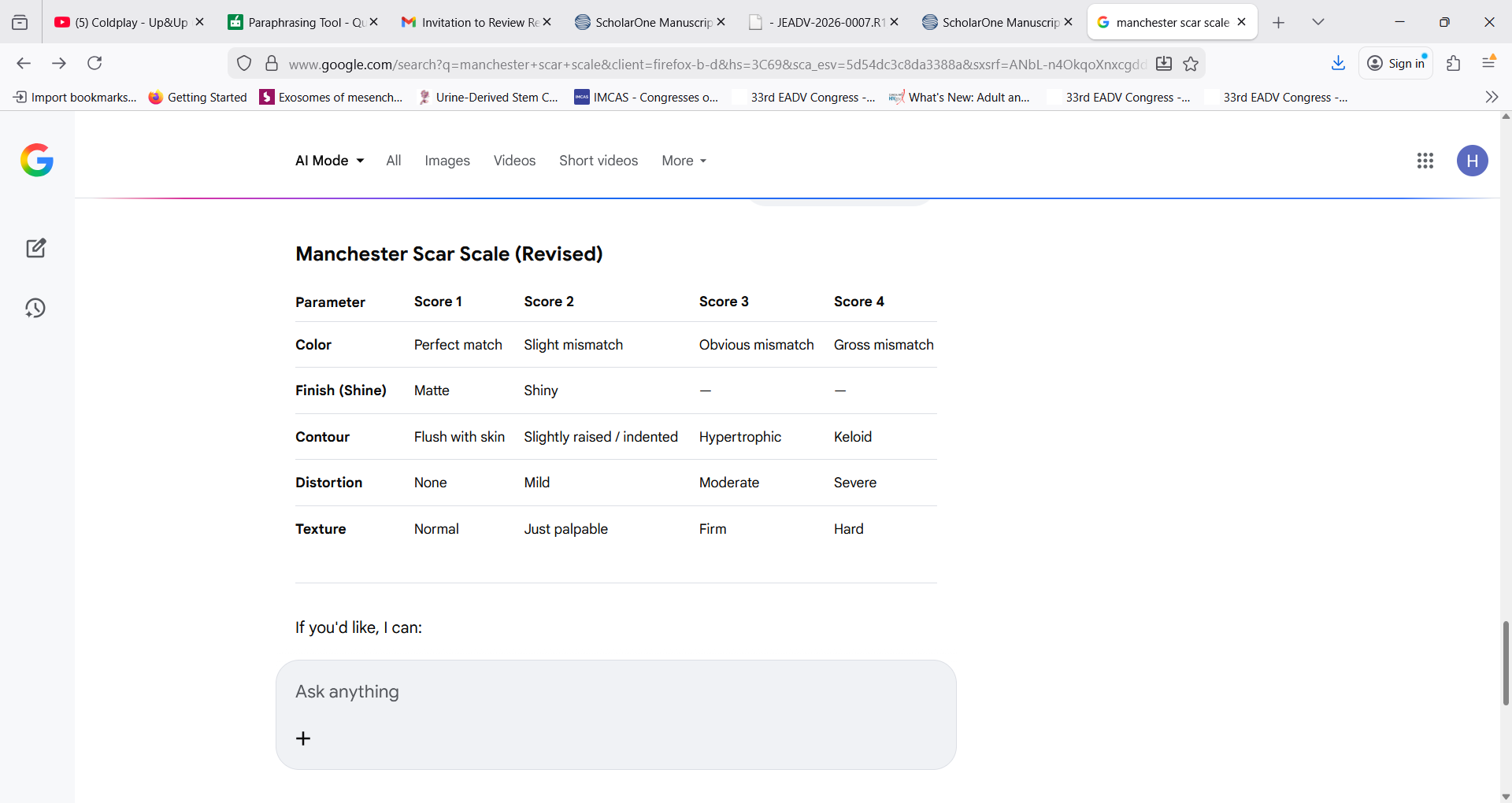
**Manchester Scar Scale**

Supplement: ojag121_Supplementary_Data [file ojag121_supplementary_data.docx]
